# The efficacy and safety of chemo‐free therapy in epidermal growth factor receptor tyrosine kinase inhibitor‐resistant advanced non‐small cell lung cancer: A single‐arm, phase II study

**DOI:** 10.1002/cam4.6545

**Published:** 2023-09-18

**Authors:** Shuyang Zhang, Lu Yang, Yaning Yang, Guangjian Yang, Haiyan Xu, Xueliang Niu, Yan Wang

**Affiliations:** ^1^ Cancer Center, Beijing Tongren Hospital Capital Medical University Beijing China; ^2^ Department of Medical Oncology and Radiation Sickness Peking University Third Hospital Beijing China; ^3^ Department of Medical Oncology, National Cancer Center/National Clinical Research Center for Cancer/Cancer Hospital Chinese Academy of Medical Sciences and Peking Union Medical College Beijing China; ^4^ Department of Respiratory Medicine, Shandong Cancer Hospital and Institute Shandong First Medical University and Shandong Academy of Medical Sciences Jinan China; ^5^ Department of Comprehensive Oncology, National Cancer Center/National Clinical Research Center for Cancer/Cancer Hospital Chinese Academy of Medical Sciences and Peking Union Medical College Beijing China; ^6^ Department of Medical Affairs Shanghai Junshi Biosciences Co., Ltd. Shanghai China

**Keywords:** antiangiogenic agents, chemo‐free therapy, *EGFR* gene mutation, immune checkpoint inhibitors, non‐small cell lung cancer

## Abstract

**Objectives:**

The purpose of this study was to explore the efficacy and safety of toripalimab combined with anlotinib in patients with advanced non‐small cell lung cancer (NSCLC) who acquired resistance to epidermal growth factor receptor tyrosine kinase inhibitors (EGFR‐TKIs).

**Materials and Methods:**

Patients who developed resistance after using first‐ or second‐generation EGFR‐TKIs as their first‐line regimen without *EGFR* T790M mutation or had disease progression after being treated with third‐generation EGFR‐TKIs as first‐ or second‐line therapy were enrolled. All patients received toripalimab (240 mg/day on Day 1, intravenously) combined with anlotinib (12 mg/day, Days 1–14, orally) once every 3 weeks. Treatment continued until disease progression, or if toxicity was intolerable. The primary endpoint was the objective response rate (ORR) assessed by the investigator. The secondary endpoint was the progression‐free survival (PFS).

**Results:**

In total, 19 patients were enrolled between May 2020 and October 2021.The ORR was 0%, and a median PFS was 2.1 months (95% CI 0.251–3.949). Grade ≥3 treatment‐related adverse events (AEs) occurred in 11% patients. Common adverse events included hypothyroidism (12/19), fatigue (9/19), and hypertension (8/19). Patients in stable disease (SD) group had lower abundance of *EGFR* mutation allele frequency (AF) before enrollment than those in progressive disease (PD) group (*p* = 0.031). Patients without detectable *EGFR* mutation (EGFR−) had longer PFS compared to the ones with *EGFR* mutations (*p* = 0.059). Patients with high levels of soluble programmed cell death ligand 1 (PD‐L1) at baseline also tended to have longer PFS (*p* = 0.160).

**Conclusion:**

Toripalimab combined with anlotinib was tolerable in EGFR‐TKI‐resistant advanced NSCLC patients not previously treated with chemotherapy. Patients without detectable *EGFR* mutation and high soluble PD‐L1 levels may benefit from this chemotherapy‐free treatment.

## 1 INTRODUCTION

Epidermal growth factor receptor (*EGFR*) gene mutations are key factors in the development of non‐small cell lung cancer (NSCLC), with an incidence of 40%–60% in Asian populations.^1^ Patients harboring *EGFR* mutations can achieve long‐term survival with EGFR‐TKIs.^2^, ^3^, ^4^, ^5^ However, acquired drug resistance is inevitable in most of these patients after several cycles of treatment. Numerous clinical trials seeking to overcome resistance to TKIs in a wide range of cancers are underway or have already been finished.^6^, ^7^

Both immune evasion and angiogenesis are important factors in tumor development.^8^ Although about 20% of patients with advanced NSCLC can benefit from immune checkpoint inhibitors (ICIs),^9^ patients with EGFR‐TKI resistance do not benefit from ICI monotherapy. The Checkmate057,^9^ KEYNOTE‐010,^10^ and OAK studies^11^ reported that EGFR‐mutated patients who received nivolumab, atezolizumab, or pembrolizumab monotherapy did not obtain a benefit in overall survival (OS).

The immune microenvironment of *EGFR* mutation‐positive patients is of the immune‐desert or immune‐excluded phenotypes,^12^, ^13^, ^14^ which may result in a poor response to immunotherapy. A great number of preclinical studies have demonstrated that immunotherapy plus chemotherapy or antiangiogenic agents have robust synergistic antitumor efficacy.^15^, ^16^ Chemotherapy or antiangiogenic agents can increase tumor‐infiltrating lymphocytes (TILs) in the tumor microenvironment and transform the immune‐desert phenotypes into the inflamed phenotypes.^17^ Since combination treatment can promote the efficacy of immunotherapy by increasing both tumor mutation burden (TMB) and tumor antigen exposure,^18^, ^19^ combination therapy has become one of the major research trends for EGFR‐TKI‐resistant patients.^20^, ^21^, ^22^ A phase II clinical trial showed that toripalimab combined with chemotherapy can provide a 50% ORR and 7.0 months of PFS for patients for whom first‐line EGFR‐TKI therapy failed.^23^

For the advanced NSCLC patients, the four‐drug regimen in the IMpower150 study in which ICIs, chemotherapy, and antiangiogenic agents were combined showed favorable efficacy as a first‐line therapy.^24^, ^25^, ^26^ Specifically, in comparison with the control group (chemotherapy combined with bevacizumab), the four‐drug combination therapy significantly prolonged PFS in patients harboring *EGFR* mutations or *ALK* translocation (9.7 vs 6.1 months, HR 0.59, 95% CI 0.37–0.94, *p =* 0.0253). Based on the final analysis of this study, the four‐drug regimen extended OS by an additional 11.3 months with chemotherapy plus bevacizumab in patients harboring *EGFR* 19 Del or *EGFR* 21 L858R.^27^ This study confirmed that immune combination therapy can bring clinical advantages to *EGFR*‐mutant patients.

However, the four‐drug regimen increased medication and the treatment expense. In clinical practice, the majority of patients who receive long‐term targeted therapy refuse subsequent chemotherapy. Therefore, whether chemo‐free therapies can benefit the survival of EGFR‐TKI‐resistant patients has also attracted much attention. Antiangiogenic therapy can normalize blood vessels, thereby allowing programmed cell death protein 1(PD‐1) inhibitors or programmed cell death ligand 1 (PD‐L1) inhibitors to enter the tumor microenvironment. On the other hand, blocking vascular endothelial growth factor (VEGF) can reduce Treg cells in the tumor microenvironment and relieve immunosuppression.^28^ In a small, single‐arm prospective clinical study led by Professor Baohui Han at Shanghai Chest Hospital, sintilimab combined with anlotinib showed significant efficacy in patients with advanced driver gene‐negative NSCLC.^29^ However, there is uncertainty about the efficacy of chemo‐free therapies in the EGFR‐TKI‐resistant population. Several retrospective studies have mentioned the potential importance of antiangiogenic therapies in the medical care of patients resistant to TKIs. To date, there are no prospective clinical studies to elucidate the efficacy and safety of chemo‐free therapies.

Therefore, in this study, we investigated the efficacy and safety of toripalimab combined with anlotinib for patients who acquired resistance to first‐, second‐, or third‐generation EGFR‐TKIs and also explored efficacy‐related biomarkers.

## 2 MATERIALS AND METHODS

### 2.1 Study design

This study was a prospective, single‐center, single‐arm phase II clinical study that was approved by the Ethics Committee of Cancer Hospital, Chinese Academy of Medical Sciences (Approval Number:19/214‐1998). The registration number of Chinese Clinical Trial Registry is ChiCTR1900028112. The study was conducted in accordance with the Declaration of Helsinki. Informed consent was signed by all patients. The response rate to second‐line chemotherapy in historical control patients with non‐squamous NSCLC was 30%, which was expected to increase to 50% in this study. Simon's two‐stage design was performed with *α* = 0.05 and *β* = 0.2. The minimum sample size was 39 patients. Nineteen patients were enrolled in the first stage. If six patients achieved partial response (PR) or complete response (CR), an additional 20 patients were recruited for the second phase of this study. Finally, if 16 patients achieved PR or CR, it was confirmed that this treatment regimen was effective.

Patients with advanced lung adenocarcinoma who visited to Cancer Hospital, Chinese Academy of Medical Sciences, from May 2020 to October 2021 were enrolled in the study. Inclusion criteria: (1) Stage IV, *EGFR*‐mutant NSCLC confirmed by pathological examination of tissue specimens or cytological specimens; (2) disease progression after first‐line treatment with first‐ or second‐generation EGFR‐TKIs and histological or blood tests after progression suggesting no T790M mutation; (3) disease progression after first‐line or second‐line treatment with third‐generation EGFR‐TKIs; (4) at least one measurable target lesion (5) age of 18–80 years old and Eastern Cooperative Oncology Group Performance Status (ECOG PS) score of 0 to 2. Exclusion criteria: (1) autoimmune disease history; (2) bleeding tendency or coagulation disorders and the first use of study drug within 4 weeks before the occurrence of CTCAE 3 and above bleeding at other sites; (3) arteriovenous thrombotic events that occurred within 2 months before the first dose; (4) previously received chemotherapy. Clinical information collected in the study included sex, age, smoking history, metastatic site, genetic testing results, and EGFR‐TKI treatment.

All enrolled patients were treated with toripalimab (240 mg intravenously [IV] on Day 1, once every 3 weeks for 4 cycles). Anlotinib was taken orally once a day continuously on the first day through the 14th day of a 3‐week cycle. In the first stage, three patients were assigned to the dose‐escalation phase of anlotinib, and the administration method in cycle one was 8 mg daily for 14 consecutive days followed by 1‐week discontinuation. If the patient did not experience intolerable adverse reactions, the daily dose was increased to 12 mg at the beginning of the second cycle. For patients who are too weak to undergo dose adjustment, the 8‐mg dose was to be maintained from cycle two. If the first three enrolled patients could successfully transitioned to the 12‐mg treatment phase, the subsequent patients were directly given the 12‐mg dose for the first dose. This regimen was maintained until patients developed disease progression or had intolerable adverse events (AEs). If the criteria of stage I are met, the study shall enter stage II; if not, the study shall not enter stage II.

### 2.2 Efficacy evaluation

Imaging examination and hematological examination were carried out for all patients before enrollment and at the end of every 2 cycles of treatment after enrollment. At baseline and after every 2 cycles of treatment, patients underwent computed tomography (CT) scans covering the neck, chest, and abdomen. Enhanced brain magnetic resonance imaging (MRI) was required for brain metastasis. Efficacy evaluations were classified as CR, PR, stable disease (SD), or progressive disease (PD) based on Response Evaluation Criteria in Solid Tumors version 1.1 (RECIST 1.1).

### 2.3 End points

The primary endpoint was the objective response rate (ORR), which was defined as the percentage of patients who achieved CR and PR after receiving treatment. The secondary endpoint was progression‐free survival (PFS), as defined as the period from the first day of treatment with toripalimab and anlotinib to discontinuation due to disease progression or intolerance to treatment. PFS2 was defined as the period from treatment after withdrawal from the group until disease progression occurred again. The overall survival (OS) was the time from the start of enrollment treatment to death or the last follow‐up. Adverse events in this study were measured on the basis of the Common Terminology Criteria for Adverse Events (CTCAE 5.0). Follow‐up continued until the last patient was out of group for 3 months.

### 2.4 Sample collection and testing

For all patients, 8–10 mL of peripheral blood was collected dynamically and preserved in EDTA anticoagulated tubes. Blood samples were taken at the following times before treatment, at the time of each efficacy evaluation and after disease progression. Next generation sequencing (NGS) was performed on the blood samples collected at baseline, efficacy evaluation, and disease progression; TMB was calculated; and plasma PD‐L1 expression was determined by MSD (more details see Appendix S1). *EGFR* positivity was defined based on the detection limit of the cfDNA assay (≥2%). For soluble PD‐L1 (sPD‐L1) analysis, a cutoff at the 70% percentile of the sPD‐L1 concentration (137.2681 pg/mL) was used to stratify patients into PD‐L1‐H or PD‐L1‐L group.

### 2.5 Statistical analysis

All statistical analyses of the data in present trail were performed by using R software version 4.1 (R Foundation for statistical computing). Differences in categorical variables were tested using the chi‐square test or Fisher's exact test, and differences in continuous non‐normally distributed variables were tested using the Mann–Whitney *U* nonparametric test. Survival data were analyzed by the Kaplan–Meier method, and differences in survival curves were compared by the log‐rank test. Hazard ratios (HR values with 95% CI) were calculated from the Cox proportional hazards model. A two‐sided *p* value of <0.05 was set as statistically significant.

## 3 RESULTS

### 3.1 Baseline clinical characteristics of patients

From May 2020 to October 2021, 19 patients were successfully recruited. All patients were diagnosed with stage‐IV adenocarcinoma of the lung by the Department of Pathology of our hospital. The final follow‐up date was June 24, 2022. T790M mutations were found in 11 of the enrolled patients after progression of first‐ or second‐generation EGFR‐TKIs; those 11 patients received osimertinib as their second‐line therapy. Six enrolled patients who were resistant to first‐line treatment with first‐ or second‐generation EGFR‐TKI therapy did not carry the T790M mutation. The remaining two patients were enrolled after PD on first‐line treatment with osimertinib. All patients harbored *EGFR* sensitive mutations: 12 with *EGFR* 19 Del, 6 with *EGFR* 21 L858R mutations, and initially diagnosed with the T790M mutations.

The median age of this cohort was 61 years. The age range was 41–79 years old. Thirteen (68.4%) patients were female, and 16(84.2%) patients had no previous history of smoking; 14 (73.7%) patients had an ECOG PS score of 0 or 1 (see Table 1 for details).

**TABLE 1:** Clinical characteristics of patients.

Clinical features	*N* (%)
Gender	
Male	6 (31.6)
Female	13 (68.4)
Age	
<61 years	8 (42.1)
≥61 years	11 (57.9)
Smoking history	
Former/ever smoker	3 (15.8)
Never smoker	16 (84.2)
ECOG PS score	
0	3 (15.8)
1	11 (57.9)
2	5 (26.3)
Metastatic site	
Lung	10 (52.6)
Bone	6 (31.6)
Brain	8 (42.1)
Adrenal gland	3 (15.8)
Liver	4 (21.1)
Pleura	8 (42.1)
Oncogene status	
*EGFR* 19 DEL	12 (63.2)
*EGFR* 21 L858R	6 (31.6)
*EGFR* T790M	1 (5.2)
Prior Therapy	
First‐line EGFR‐TKI	
Icotinib	6 (31.6)
Dacomitinib	4 (21.1)
Erlotinib	1 (5.2)
Gefitinib	6 (31.6)
Osimertinib	2 (10.5)
Second‐line EGFR‐TKI	
Osimertinib	11 (57.9)

### 3.2 Efficacy analysis

**FIGURE 1:**
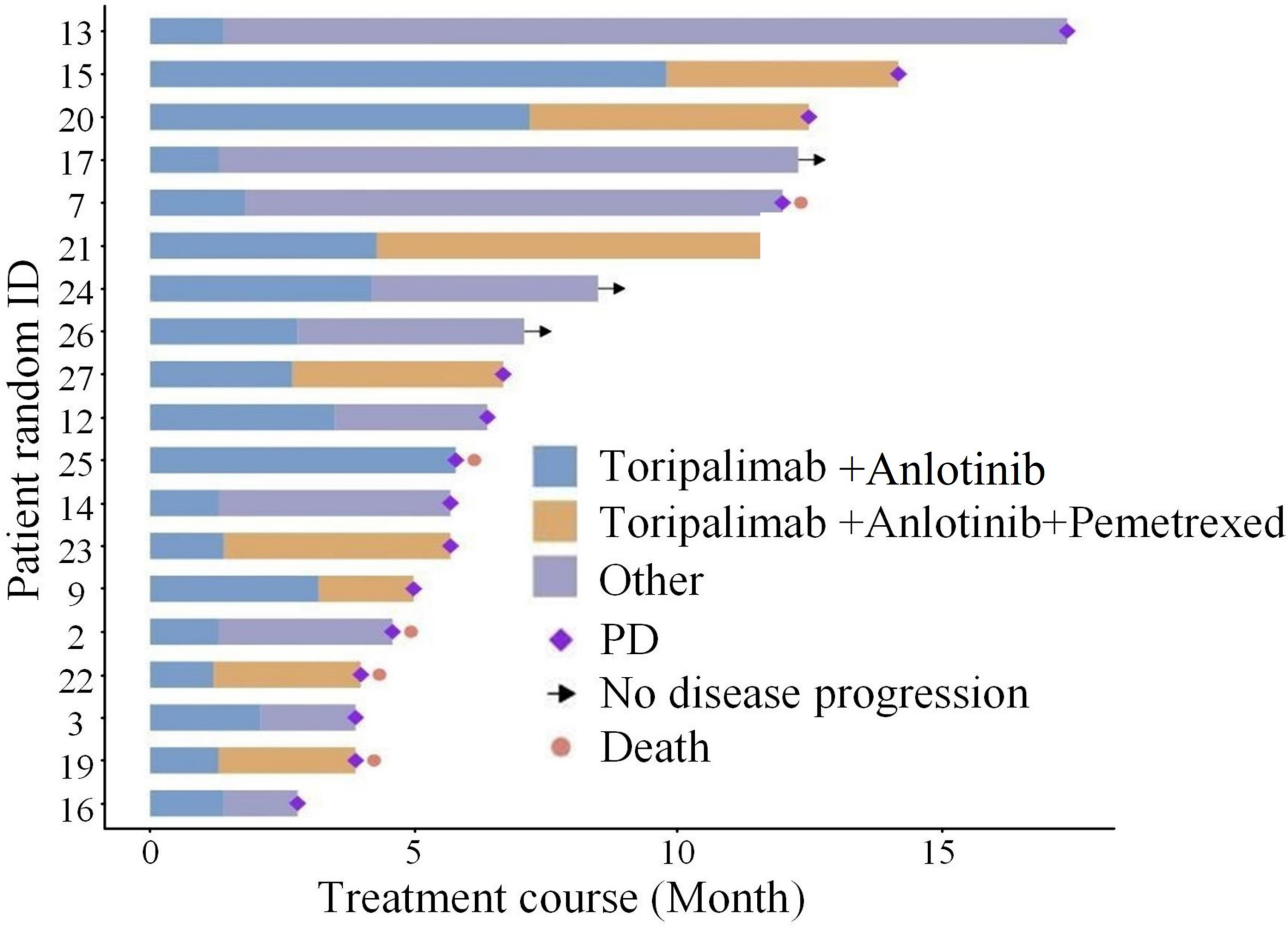
Treatment and survival time of enrolled patients.

The treatment and survival of all patients were shown in Figure 1. No patient achieved CR or PR for best response evaluation. Eleven patient (57.9%) had SD as the best response evaluation (57.9%); the other patients (8/19) showed disease progression on imaging after 2 cycles of therapy. The ORR was 0%, and the DCR was 57.9%. The median PFS was 2.1 months (95% CI 0.251–3.949).

**FIGURE 2:**
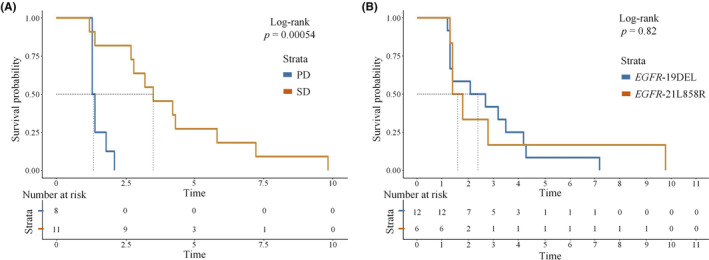
Progression‐free survival curves for different groups of patients. (A) Progression‐free survival curves for SD or PD groups; PD, progressive disease; SD, stable disease. (B) Progression‐free survival curves for patients with *EGFR* 19DEL or *EGFR* 21L858R mutations.

Compared to patients with PD, patients who obtained SD had a PFS approximately 2 months longer (3.5 vs 1.4 months, *p =* 0.0005, HR 0.104, 95% CI 0.0021–0.521) as shown in Figure 2A. PFS was slightly longer in patients initially diagnosed with *EGFR* 21 L858R than with *EGFR* 19 Del (median PFS 3.1 and 1.6 months, HR 0.579, 95% CI 0.209–1.605), but the difference was not significantly significant. The PFS of patients with *EGFR* 21L858R and *EGFR* 19 DEL is shown in Figure 2B.

### 3.3 Treatment‐related adverse events

Treatment‐related grade 3 or higher AEs occurred in 11% (2/19) of patients. The AEs are listed in Table 2, including hypothyroidism (12/19), fatigue (9/19), hypertension (8/19), transaminase elevation (8/19), proteinuria (4/19), oral ulcer (4/19), hand‐foot syndrome (4/19), and hoarseness (3/19). Patients with grade I–II AEs recovered after symptomatic treatment without dose adjustment, though dose adjustment was performed for two patients with grade III AEs: one who previously received 6 cycles of treatment discontinued anlotinib due to hypertension; one for whom the dose was adjusted to 8‐mg anlotinib after 3 cycles of treatment due to severe hoarseness, and the disease progressed after the end of the fourth cycle of treatment.

**TABLE 2:** Treatment‐related adverse events.

Adverse events	Number (%)					
	All	Grade I	Grade II	Grade III	Grade IV	Grade V
Neutropenia	2 (11)	2 (11)	0	0	0	0
Anemia	1 (5)	1 (5)	0	0	0	0
Thrombocytopenia	1 (5)	1 (5)	0	0	0	0
Asthenia	9 (47)	7 (37)	2 (11)	0	0	0
Rash	2 (26)	1 (5)	1 (5)	0	0	0
Hypertension	8 (42)	4 (21)	3 (16)	1 (5)	0	0
Proteinuria	4 (21)	3 (16)	1 (5)	0	0	0
Transaminases increased	8 (42)	7 (37)	1 (5)	0	0	0
Hoarseness	3 (16)	0	2 (11)	1 (5)	0	0
Epistaxis	2 (11)	2 (11)	0	0	0	0
Hemoptysis	2 (11)	1 (5)	1 (5)	0	0	0
Pneumonia	2 (11)	0	2 (11)	0	0	0
Pneumothorax	2 (11)	1 (5)	1 (5)	0	0	0
Hypothyroidism	12 (63)	8 (42)	4 (21)	0	0	0
Oral ulcer	4 (21)	3 (16)	1 (5)	0	0	0
Hand‐foot syndrome	4 (21)	3 (16)	1 (5)	0	0	0

### 3.4 Exploratory analyses

**FIGURE 3:**
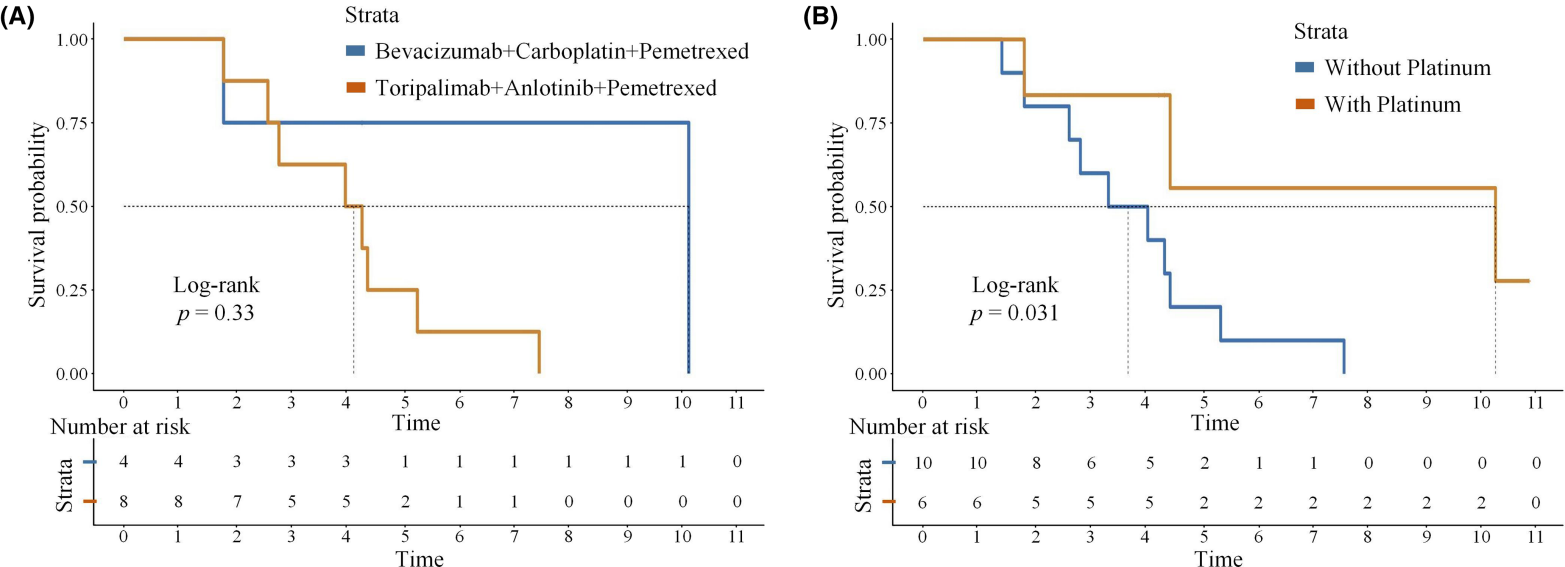
Progression‐free survival curves for patients with different treatment. (A) Progression‐free survival curves 2 for patients with bevacizumab, carboplatin, pemetrexed or toripalimab, anlotinib, and pemetrexed. (B) Progression‐free survival curves 2 for patients with or without platinum.

Eighteen patients received different regimens after dropout. Eight patients received the original regimen (toripalimab plus anlotinib) in combination with pemetrexed, four patients used pemetrexed and carboplatin in combination with bevacizumab, and one patient used pemetrexed and bevacizumab plus sintilimab; the remaining patients' regimens are shown in the Table S1. The median PFS2 of the original regimen plus pemetrexed compared with pemetrexed, carboplatin, and bevacizumab was 4.2 months and 10.2 months, respectively (log‐rank *p* = 0.33; see Figure 3A for details). Continuation of the original regimen combined with pemetrexed after withdrawal from the group increased the risk of disease progression (HR = 3.026, 95% CI 0.7502–12.2). The median PFS2 for the later line platinum‐containing chemotherapy versus platinum‐free chemotherapy was 10.2 and 3.7 months, respectively. The platinum‐containing regimen significantly reduced the risk of disease progression (HR = 0.2683, 95% CI 0.083–0.86) as detailed in Figure 3B.

**FIGURE 4:**
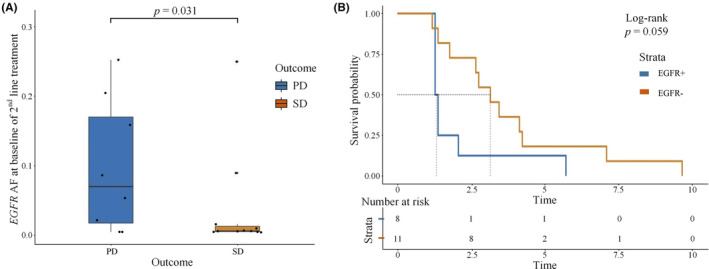
Relationship between baseline *EGFR* AF and efficacy. (A) EGFR AF at baseline in different group; PD, progressive disease; SD, stable disease. (B) Progression‐free survival curves for patients with or without *EGFR* mutation baseline; EGFR−, EGFR negativeness; EGFR+, EGFR positiveness.

**FIGURE 5:**
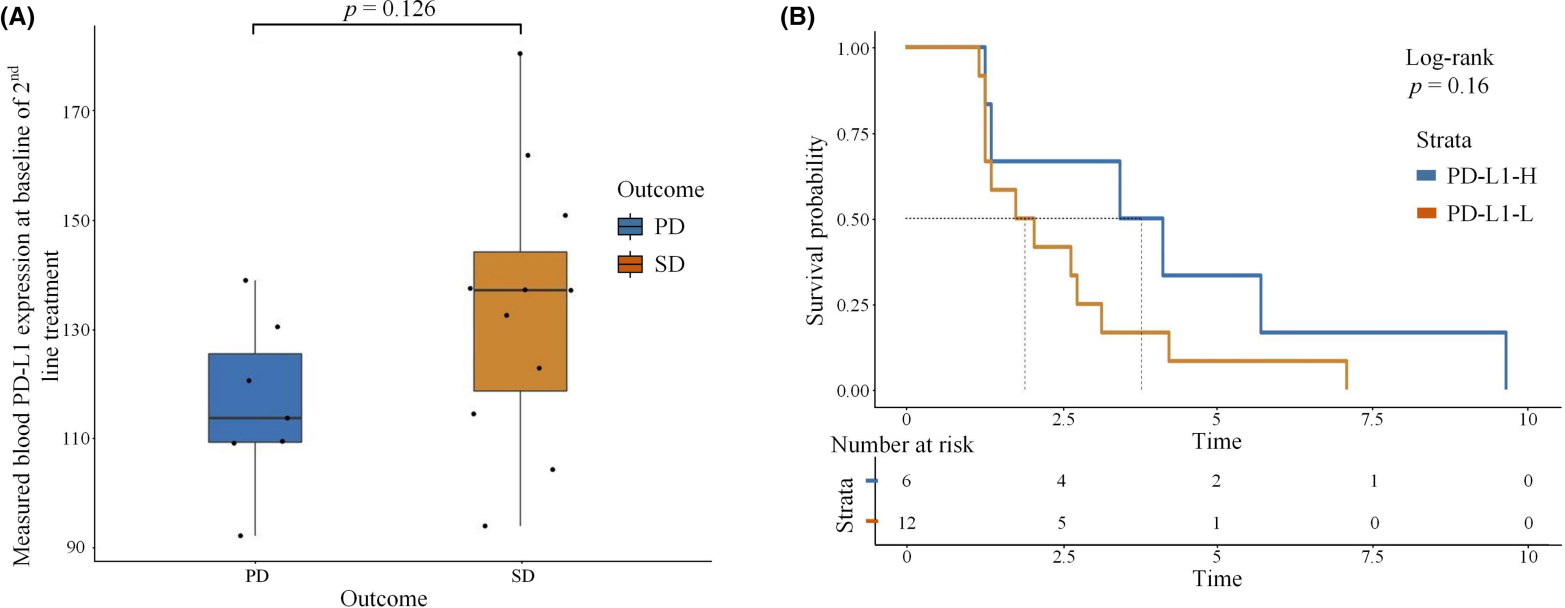
Relationship between baseline blood soluble PD‐L1 concentration and efficacy. (A) blood soluble PD‐L1 expression at baseline in different group; PD, progressive disease; SD, stable disease; (B) Progression‐free survival curves for patients with high level or low level of blood soluble PD‐L1 expression at baseline; PD‐L1‐H, high level of blood soluble PD‐L1 expression at baseline; PD‐L1‐L, low level of blood soluble PD‐L1 expression at baseline.

Of the 206 mutations detected in the patients' peripheral blood samples, *EGFR* mutations showed the highest frequency (see Figure S1 for details). The difference in ctDNA score at baseline as well as before and after treatment was associated with efficacy (see Figures S2 and S3 for details). Patients in the SD group had a lower baseline *EGFR* allele frequency (AF) than those in the PD group (*p* = 0.031). Patients without detectable *EGFR* mutation (EGFR−) had longer PFS than those with *EGFR* mutations (see Figure 4A,B for details). Compared with patients in PD group, patients in SD group tend to have higher blood concentrations of soluble sPD‐L1 in baseline (*p* = 0.126; see Figure 5A for details). There was also a tendency for patients with high sPD‐L1 levels to have prolonged PFS compared to those with low sPD‐L1 levels, but it was not statistically significant (*p* = 0.16; see Figure 5B for details). No differences were found in bTMB between the SD and PD groups (as detailed in Figure S4).

## 4 DISCUSSION

This study investigated the feasibility of toripalimab plus anlotinib for patients resistant to EGFR‐TKI. In present study, the intent‐to‐treat (ITT) population did not benefit from the chemo‐free therapies. However, some patients, such as those patients without detectable *EGFR* mutation and high level of sPD‐L1 at baseline, did benefit from the toripalimab combined with anlotinib.

Chemo‐free therapies such as ICIs combined with antiangiogenic therapy, and ICIs combined with targeted therapy are being explored in EGFR‐TKI‐resistant populations. Current guidelines do not recommend this combination regimen due to the severe adverse effects of ICI combined with targeted therapy, which are difficult for patients to tolerate.^30^, ^31^ For ICIs combined with antiangiogenic therapy, Cai‐Cun Zhou's team assessed the efficacy of this therapeutic schedule in a phase II multicohort clinical trial. The ORR of apatinib combined with camrelizumab in EGFR‐TKI‐resistant patients at the later line was only 18%, with a median PFS of 2.8 months.^32^ This result is similar to that of the present study. However, some of the patients enrolled in Zhow's clinical trial had received prior systemic chemotherapy; therefore, the results of the study may be influenced by prior chemotherapy. As the population enrolled in our study had not previously received systemic chemotherapy, so the results may be more representative. At the World Conference on Lung Cancer (WCLC) 2022, a report of a clinical study with a small sample size showed that the ORR of sintilimab combined with anlotinib in patients with rare *EGFR* mutations (*EGFR* 20 ins, L861Q, G719A, and G709T) who had previously been treated with targeted therapy or chemotherapy was 36.8%, with a median PFS of 6.7 months. Previous retrospective studies had also found better efficacy for immunotherapy in rare EGFR‐mutant patients compared to those with common EGFR mutations.^33^ In contrast to patients with common *EGFR* mutations, patients with rare *EGFR* mutations have higher PD‐L1 expression^34^ and TMB levels.^35^ Differences in the tumor microenvironment may be the reason why patients with different subtypes of *EGFR* mutations respond disparately to ICIs. All the patients enrolled in the present research had common *EGFR* mutations.

The tested regimen was well tolerated in our cohort. There were no new safety issues identified, and AEs were similar to those described in previous clinical studies.^36^, ^37^ Mild hypothyroidism, hypertension, elevated transaminases, and other adverse reactions can be controlled by clinical evaluation and appropriate treatment. Two cases of spontaneous asymptomatic pneumothorax occurred, which was interpreted to be associated with the patient's intrapulmonary metastases close to the pleura and cavities formed after treatment with anlotinib.

In both Impower150 and ORIENT‐31 studies,^38^ chemotherapy combined with antiangiogenic therapy and ICIs led to important discoveries in patients resistant to EGFR‐TKI. An interim analysis of the ORIENT31 study showed that a four‐drug regimen resulted in an ORR of 44% and a median PFS of 6.9 months in EGFR‐TKI‐resistant patients. Moreover, a 50% ORR was achieved in T790M‐negative patients after resistance to EGFR‐TKIs by toripalimab combined with chemotherapy.^23^ In our study, a significant prolongation of PFS2 was found in patients receiving late‐line platinum‐based therapy. In general, a significant increase in the risk of disease progression is associated with platinum‐free chemotherapy. This suggests that platinum‐based doublet chemotherapy may be an essential component for treatment of driver gene‐positive patients. We speculate that this may be associated with the fact that patients with *EGFR* mutations have a desert immunophenotype and chemotherapy may increase the release of antigens, which is associated with increased efficacy of immunotherapy.

The effective population of ICIs plus antiangiogenic drugs in patients with advanced EGFR‐TKI‐resistant NSCLC requires further exploration. In *EGFR*‐mutant patients who had undergone targeted therapy and previous chemotherapy, the ORR of apatinib combined with camrelizumab was only 18% and the median PFS of 2.8 months. Furthermore, in the above‐mentioned study, ORR and PFS were higher in *EGFR* L858R patients than in *EGFR* 19Del patients.^32^ Similar results were observed in our study, with a median PFS of 2.1 months, and a slightly longer median PFS for *EGFR* L858R‐mutant patients than for *EGFR* 19 DEL patients. It has previously been shown that patients with *EGFR* L858R had more CD8^+^ T‐cell infiltration than patients with *EGFR* 19Del.^39^ Tumor heterogeneity is higher in patients with the L858R than those with *EGFR* 19Del, which may explain the difference in PFS between these two subtypes.

In present study, blood ctDNA was detected in baseline and after disease progression in all patients. Several clinical studies have demonstrated that high ctDNA abundance was associated with worse prognosis,^40^, ^41^ and increased ctDNA during treatment leads to worse response to immunotherapy.^42^, ^43^ Unfortunately, we did not find a correlation between dynamic changes in ctDNA and efficacy, which may be associated with the small size of the cohort and the fact that few patients achieved significant clinical remission. Surprisingly, we observed that lower baseline *EGFR* mutation abundance in SD patients. Patients without detectable EGFR mutation (EGFR‐) had longer PFS compared to the ones with EGFR mutations. Retrospective studies have found that changes in *EGFR* abundance are associated with prognosis.^44^, ^45^, ^46^ The BENEFIT study^47^ prospectively demonstrated that clearance of *EGFR* from ctDNA predicted longer PFS. We speculate that the low *EGFR* abundance after TKI treatment may indicate that more *EGFR*‐mutated tumor cells are removed, fewer tumor cells are resistant to immunotherapy, and residual tumor cells are more likely to respond to ICIs. Furthermore, sPD‐L1 concentration was higher in patients who archived SD. Whether the sPD‐L1 can serve as a predictor for the efficacy of immunotherapy is unclear. Soluble PD‐L1 has been demonstrated to be a poor prognostic factor for immunotherapy in NSCLC.^48^, ^49^, ^50^ However, a small study in *EGFR*‐positive patients observed that posttreatment increases in soluble PD‐L1 are associated with better PFS with TKI treatment.^47^ To date, no studies have explored the association between sPD‐L1 and immunotherapy efficacy in *EGFR*‐mutant patients, and more data are needed to support whether soluble PD‐L1 can predict immunotherapy efficacy in these patients.

Although this was a prospective study, some limitations must be mentioned. First, given the small size of sample, the results need to be carefully understood and validated by large sample‐size studies. Furthermore, considering the difficulty of obtaining tissue samples from patients before enrollment and after disease progression, it was impossible to further detect and explore the changes in the tumor microenvironment.

## 5 CONCLUSIONS

Toripalimab combined with anlotinib was tolerable in patients with EGFR‐TKI‐resistant advanced NSCLC who had not previously received chemotherapy, but this combination model therapy did not achieve the expected efficacy. Patients without detectable *EGFR* mutation and high sPD‐L1 levels may benefit from this therapy, but this finding remains to be further explored and validated. Further exploration of feasible regimens in chemo‐free therapy is still necessary for EGFR‐TKI‐resistant patients.

## AUTHOR CONTRIBUTIONS

**Shuyang Zhang:** Data curation (equal); formal analysis (equal); investigation (equal); methodology (equal); project administration (equal); software (equal); supervision (equal); validation (equal); visualization (equal); writing – original draft (equal); writing – review and editing (equal). **Lu Yang:** Data curation (equal); formal analysis (equal); investigation (equal); methodology (equal); validation (equal); writing – review and editing (equal). **Yaning Yang:** Formal analysis (equal); methodology (equal); resources (equal); software (equal). **Guangjian Yang:** Data curation (equal); formal analysis (equal); validation (equal). **Haiyan Xu:** Resources (equal); software (equal); validation (equal). **Xueliang Niu:** Data curation (equal); methodology (equal); validation (equal). **Yan Wang:** Conceptualization (equal); formal analysis (equal); funding acquisition (equal); investigation (equal); resources (equal); supervision (equal); visualization (equal); writing – review and editing (equal).

## CONFLICT OF INTEREST STATEMENT

The authors declare no conflict of interest.

## ETHICS STATEMENT

The present study was carried out based on the Declaration of Helsinki and approved by the Institutional Review Board of the National Cancer Center/National Clinical Research Center for Cancer/Cancer Hospital, Chinese Academy of Medical Sciences and Peking Union Medical College (No.19–214/1988).

## PATIENT CONSENT STATEMENT

Written informed consent was obtained from the individuals involved in the study for the publication of this article.

## CLINICAL TRIAL REGISTRATION NUMBER

The registration number of Chinese Clinical Trial Registry is ChiCTR1900028112.

## Supporting information


Appendix S1.

## Data Availability

Data supporting the conclusions of this study are provided in the paper and its additional files. Further inquiries can be directed to the corresponding author.
