# Supplementary material for: The efficacy and safety of chemo‐free therapy in epidermal growth factor receptor tyrosine kinase inhibitor‐resistant advanced non‐small cell lung cancer: A single‐arm, phase II study
Source: Cancer Med. 2023 Sep 18;12(19):19438–48. doi: 10.1002/cam4.6545 (PMC10587943; doi:10.1002/cam4.6545)
Supplement: Supplementary file 1 — Appendix S1. [file CAM4-12-19438-s001.docx]

# Appendix：


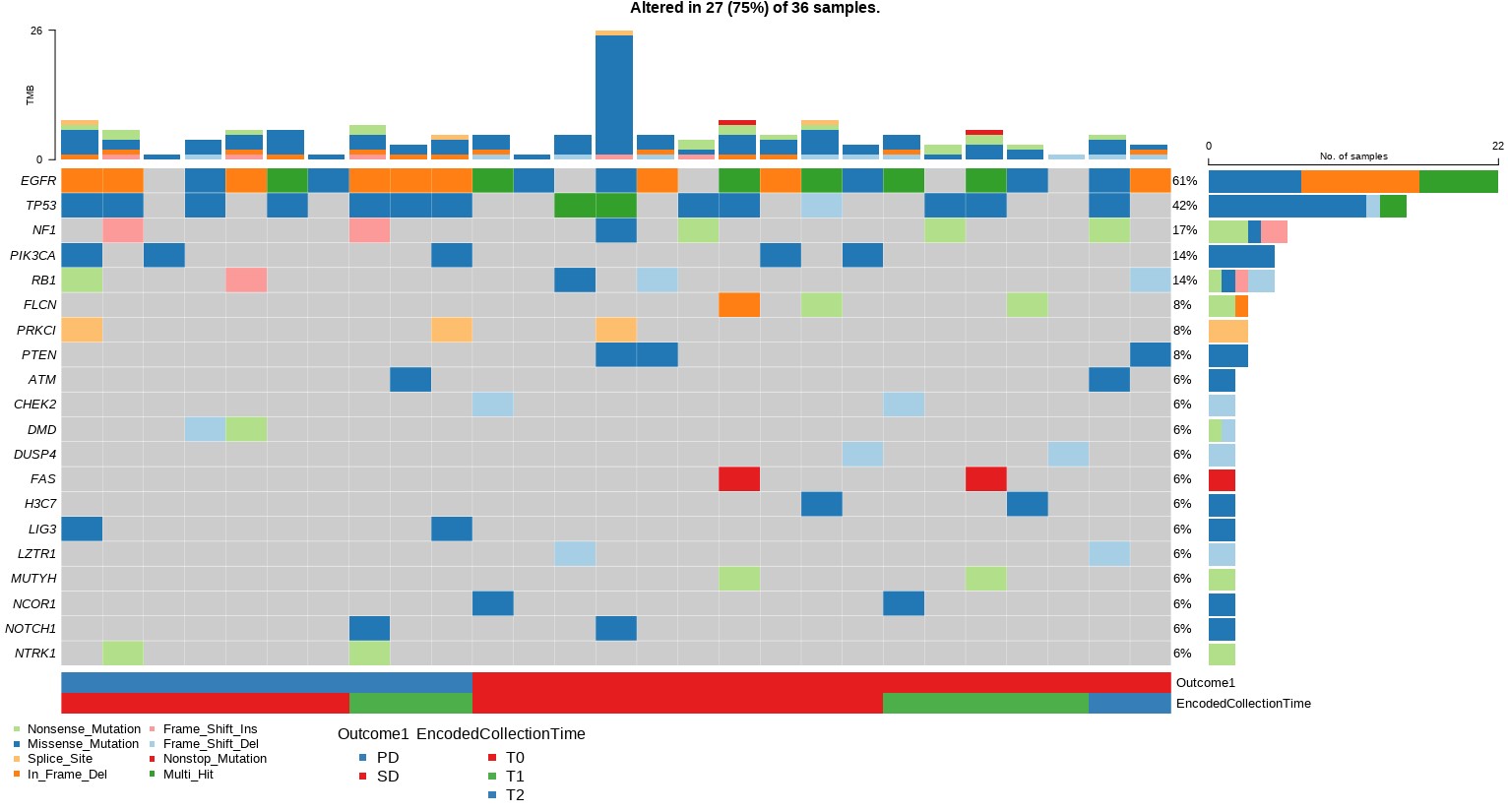


# Fig.S1. Patient Gene Mutation Heatmap


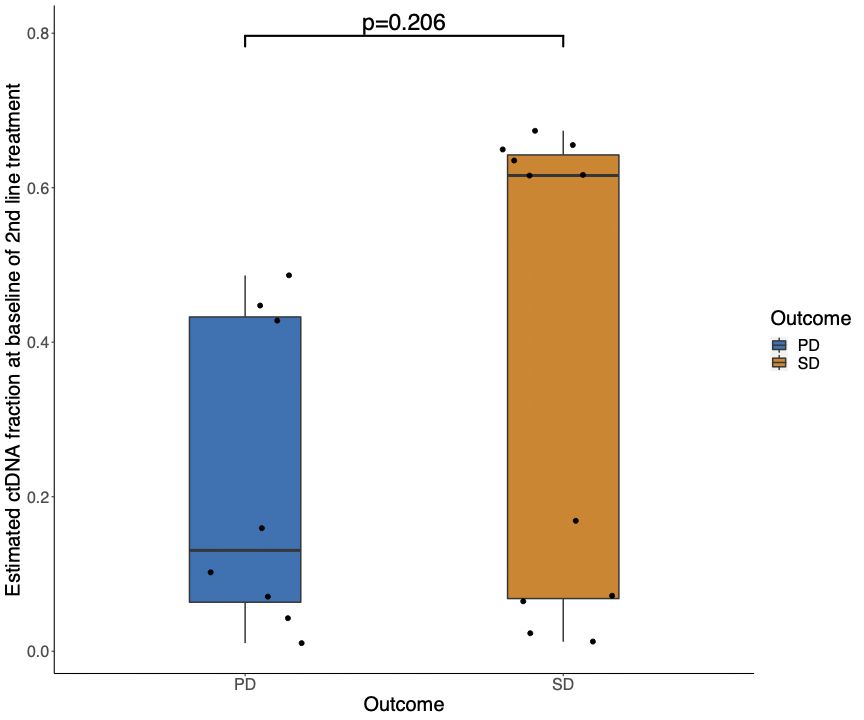


# Fig.S2. Relationship between baseline ctDNA and efficacy. PD, progressive disease; SD, stable disease


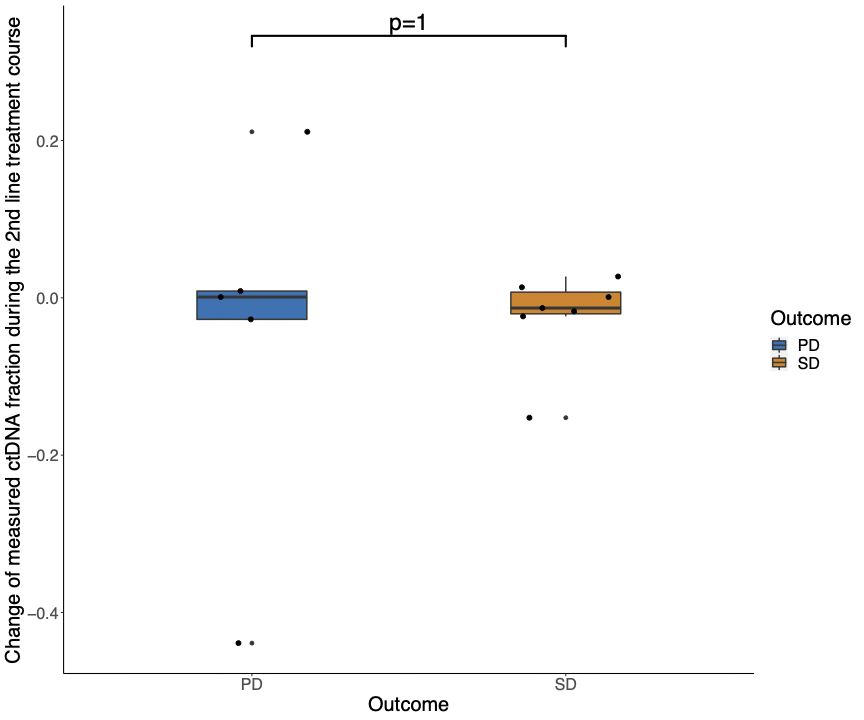


# Fig.S3. Correlation between the difference of ctDNA before and after treatment and efficacy. PD, progressive disease; SD, stable disease


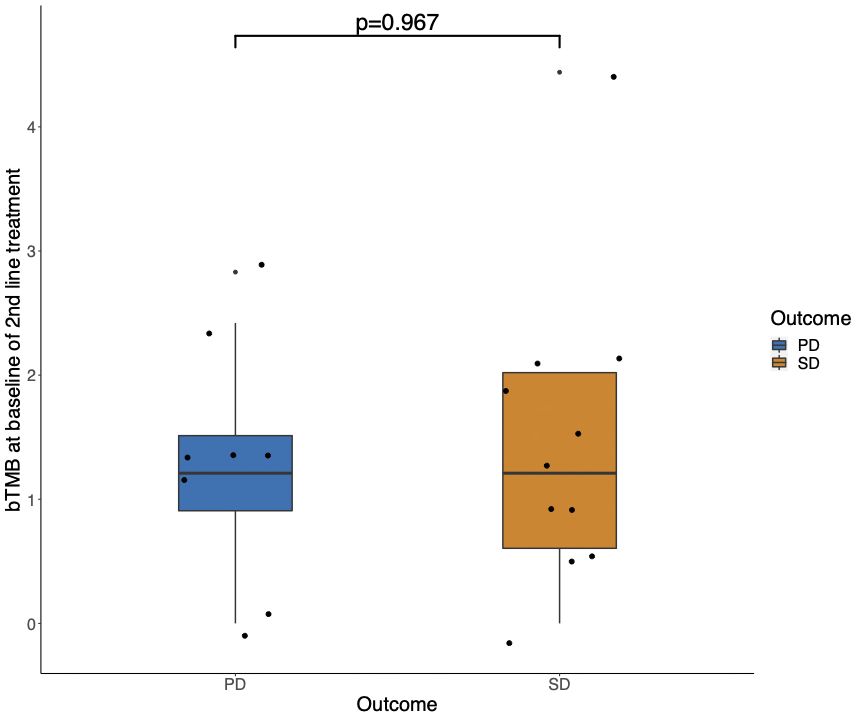


# Fig.S4. Relationship between baseline blood bTMB and efficacy. PD, progressive disease; SD, stable disease

# Table S1. Post-Withdrawal Regimens

| **Random No.** | **Gender** | **Post-progression Regimen** | **PFS2** |
| --- | --- | --- | --- |
| 2 | Male | Pemetrexed + bevacizumab +sintilimab | 3.3 |
| 3 | Female | Pemetrexed + carboplatin + bevacizumab | 1.8 |
| 7 | Female | Pemetrexed + carboplatin + bevacizumab | 10.2 |
| 9 | Female | Toripalimab + anlotinib + pemetrexed | 1.8 |
| 12 | Female | Osimertinib | 2.9 |
| 13 | Male | Osimertinib | 16.0 |
| 14 | Male | Pembrolizumab + pemetrexed + carboplatin | 4.4 |
| 15 | Female | Toripalimab + anlotinib + pemetrexed | 4.4 |
| 16 | Female | Pemetrexed + bevacizumab | 1.4 |
| 17 | Male | Atezolizumab+ pemetrexed + carboplatin + bevacizumab | 11.0 |
| 19 | Female | Toripalimab + anlotinib + pemetrexed | 2.6 |
| 20 | Female | Toripalimab + anlotinib + pemetrexed | 5.3 |
| 21 | Female | Toripalimab + anlotinib + pemetrexed | 7.5 |
| 22 | Male | Toripalimab + anlotinib + pemetrexed | 2.8 |
| 23 | Female | Toripalimab + anlotinib + pemetrexed | 4.3 |
| 24 | Female | Pemetrexed + carboplatin + bevacizumab | 4.3 |
| 26 | Female | Pemetrexed + carboplatin + bevacizumab | 4.3 |
| 27 | Male | Toripalimab + anlotinib + pemetrexed | 4.0 |

# Supplementary Materials:

**MSD Assay for PD-L1**

(a) Place the detection reagent and samples at room temperature, and take out the required well plate.

(b) Reagents, standards, and samples to be tested were prepared according to the U-PLEX Human PD-L1 (Epitope) Assay kit instructions and diluted to establish an appropriate concentration gradient.

(c) Operate in the order of coating, plate washing, adding samples or standards, plate washing, adding detection antibodies, and plate washing. Finally, place the plate into a high-sensitivity multi-factor electrochemiluminescence analyzer (SECTOR S 600).

**NGS and TMB calculation**

(a) 4 ml of plasma was used to extract cf/ctDNA using the QIAamp Circulating Nucleic Acid Kit.

(b) cf/ctDNA was quantified using a Qubit3.0 fluorescence quantifier, and fragment sizes of cfDNA/ctDNA were detected using Agilent 2100 Bioanalyzer.

(c) Library construction and hybridization operations were performed using the NEB Next Ultra II DNA Library Prep Kit for Illumina library construction kit and the MedlCDx Panel.

(d) After the completion of library construction, the concentration was detected using a Qubit 3.0 fluorescence quantifier, and the fragment size of the constructed library was detected using an Agilent 2100 Bioanalyzer.

(e) Library mixing operation. Dilute the library to the same molar concentration and mix at the same volume ratio according to the proportion of data volume.

(f) DNA sequencing was performed using an Illumina NovaSeq6000 high-throughput sequencer.

(g) Bioinformatics analysis:

Quality control of fastq data was performed using fastp software. The post-quality control data was compared to the hg19 genome to generate bam files by BWA software. The samtools s and picard software were used to sort and label bam files. The samtools was used to analyze the files to obtain mpileup files. And the mpileup files were transformed into vcf file using varsan software. Analyzed with reference to public databases to filter hotspot mutations, truncating mutations of tumor suppressor genes. Synonymous mutations and non-synonymous mutations in coding regions with maximum allele frequency (MAF) exceeding 4% were selected to calculate TMB. Calculate ctDNA fraction according to the following formula.

$$ctDNA fraction=\frac{2}{(\frac{1}{MAF}+1)}$$
